# Electroencephalographic monitoring of anesthesia during surgical procedures in mice using a modified clinical monitoring system

**DOI:** 10.1007/s10877-023-01052-y

**Published:** 2023-07-18

**Authors:** Leesa Joyce, Alissa Wenninger, Matthias Kreuzer, Paul S. García, Gerhard Schneider, Thomas Fenzl

**Affiliations:** 1 Department of Anesthesiology & Intensive Care, School of Medicine, Technical University of Munich, Munich, Germany; 2 Department of Anesthesiology, Vagelos College of Physicians and Surgeons, Columbia University, New York, NY 10032 USA

**Keywords:** Mouse, Rodents, Surgery, Anesthesia, EEG-based patient monitoring system, 3Rs

## Abstract

Monitoring brain activity and associated physiology during the administration of general anesthesia (GA) in mice is pivotal to guarantee postanesthetic health. Clinically, electroencephalogram (EEG) monitoring is a well-established method to guide GA. There are no established methods available for monitoring EEG in mice (*Mus musculus*) during surgery. In this study, a minimally invasive rodent intraoperative EEG monitoring system was implemented using subdermal needle electrodes and a modified EEG-based commercial patient monitor. EEG recordings were acquired at three different isoflurane concentrations revealing that surgical concentrations of isoflurane anesthesia predominantly contained burst suppression patterns in mice. EEG suppression ratios and suppression durations showed strong positive correlations with the isoflurane concentrations. The electroencephalographic indices provided by the monitor did not support online monitoring of the anesthetic status. The online available suppression duration in the raw EEG signals during isoflurane anesthesia is a straight forward and reliable marker to assure safe, adequate and reproducible anesthesia protocols.

## Introduction

The enhancement of translational research is still impossible without the use of animal models. The mouse not only is a well-established model organism but it is also considered a standardized translational device because of the ease of genetic manipulations mirroring human diseases [1]. Mice account for the largest proportion of mammalian species used in research [2]. The European Union alone used more than 18 million animals in 2017 and 2018 for research and substance testing, of which mice comprise 56.7% [3–5]. During the same period, an estimated 111.5 million rats and mice were used per year in the United States of America [6]. Despite the enormous number of mice used in translational studies including surgical procedures under GA, methods to assure safe, adequate and reproducible anesthesia protocols are still lacking.

In humans, pre- and intraoperative physiological assessments along with anesthetic monitoring help to ensure that a patient is safely guided through surgery with GA. Excessive or inadequate anesthesia can lead to perioperative complications such as intraoperative awareness [7, 8] or perioperative neurocognitive disorders [9–11]. As in humans, postoperative cognitive impairments, like delirium [11], can also occur in mice after anesthesia [12–14] potentially interfering with postoperative behavioral experiments and cognitive testing strategies. Although these issues are typically associated with the delivery of unnecessarily high anesthetic concentrations, other problems (i.e., stress and nociception) can occur if insufficient amounts of analgo-sedative agents are administered [15]. Physiological and pharmacological optimization of an anesthetic regime is a prerequisite for minimizing experimental variability and for maximizing safety [16].

In animal research, monitoring physiological parameters is commonly used to assess the general health status of the animal [17, 18]. Evaluation of breathing patterns, coloration of skin/mucous membranes and movement/ambulation characteristics, can also be helpful in determining the appropriate anesthetic dose. Typically, these clinical signs are also used to evaluate when the animal has recovered from surgery with GA and has returned to its baseline health status [17]. The most commonly used qualitative measures to assess anesthetic loss of responsiveness in rodents are the righting reflex [19–21], tape test [22], noxious stimuli such as the pedal withdrawal reflex in the forelimbs and hindlimbs [23–25], the tail pinch reflex [26–28] and the corneal reflex [29]. Intraoperative quantitative monitoring of physiological data in research with rodents is rarely used but specialized devices or modification of devices intended for patients can measure blood pressure, heart rate, oximetry and capnometry [30].

Though there are no established methods to monitor EEG in mice during surgery, clinical monitoring of human EEG has been facilitated by applying noninvasive EEG sensors transmitting information to an intraoperative brain function monitor. The bispectral index (BIS™ index, Medtronic, Minneapolis, USA) among others [31, 32] is the most commonly used and extensively validated EEG monitor in use by anesthesiologists [33]. The BIS™ index integrates several EEG parameters into one variable which ranges from 0 (isoelectric EEG) to 100 (wakefulness). BIS™ indices between the range of 40 to 60 are considered adequate during surgery. Along with the display of calculated indices, the real-time raw EEG signal also provides information to the surgeon [33]. This study aims to establish an easily applicable and reliable tool to ensure subject specific intraoperative monitoring during GA in mice using a modified clinical monitoring system.

## Materials and methods

### Animals

Nine adult (male, 12–18 weeks, BW: 24–27 g) wild type mice (C57BL/6N, Charles River Laboratories GmbH, Germany) were used in this study. The mice were housed individually under a 12/12-h light/dark cycle (lights on: 9am, 22 °C ± 1 °C, humidity: 55% ± 5%) with ad libitum access to food and water. All experimental procedures were approved by the Committee of Animal Health and Care of the State of Upper Bavaria, Germany (ROB-55.2–2532.Vet_02–19–121).

### EEG monitor and electrodes

For this study, a BIS™ Complete 2-channel Monitoring System (Covidien Deutschland GmbH, Germany), and subdermal needle electrodes (3 mm × 40 mm, 0.5″ × 27G (Xi’an Friendship Medical Electronics Co., Ltd.) along with a custom-made electrode-monitor interface were used. The custom-made interface was prepared as follows: The BIS™ Quatro Sensors (Covidien Deutschland GmbH, Germany) were horizontally cut at 7 cm from the microchip to expose the conducting silver/silver-chloride array. The terminal wire endings of the subdermal needles were soldered to the exposed array using gold wires (diameter: 150 µm, standard gold wire, round, which provided a stable but pliable contact) covered with soldering tin. The individual sensor interfaces were electrically insulated using heat shrink insulation tubes. Finally, the subdermal needles were electrically insulated using insulation tubes only to retain a 5 mm long conducting tip.

### Monitor settings

BIS™ monitor was used with the “filters off” setting, i.e. a bandwidth of 0.25–100 Hz. The additional filters were manually turned off as described in the user manual of the BIS™ monitor. Adequate electrode impedances were automatically measured by the BIS™ device. Small impedance offsets were corrected by delicate adjustments of the electrode placements or electrode angles on the skull. After verifying suitable impedance through all the electrodes, raw EEG signals from two channels were displayed on the monitor. Since the channel(s) to be displayed were not opted manually, the monitor automatically displayed the EEG signals from BIS™ sensor number 1. With a presetting to “export” the signals on the monitor, the displayed EEG signals were saved as R2A files (.r2a-format) to an external USB flash drive attached to the monitor with a sampling rate of 128 Hz. The trend data of the processed EEG indices was also stored to the USB flash drive as SPA (text file: .spa-format) with a resolution of one index per second.

### Anesthesia protocol

The mice were placed in a plexiglas box (Induction chamber- 8329001, AgnTho’s AB, Sweden) with approximately 1.8% isoflurane (CP Pharma, Germany) in the breathing air. After loss of responsiveness (LOR), which was verified by gently tilting the box, mice were transferred to a stereotactic frame (prone position) with an initial maintenance concentration of 1.2% isoflurane (flowrate: 192 ml/min) through an anesthesia unit, especially adapted for mice (Univentor 410 Anaesthesia unit, AgnTho’s AB, Sweden). Eye cream (Bepanthen®-Bayer AG, Germany) was applied to the eyes to avoid irritations. An automatic heating pad (Homeothermic Monitoring System, Harvard Apparatus, USA) was placed under the mouse abdomen to maintain a body temperature of 37 °C.

### Recording scenario

**Fig. 1:**
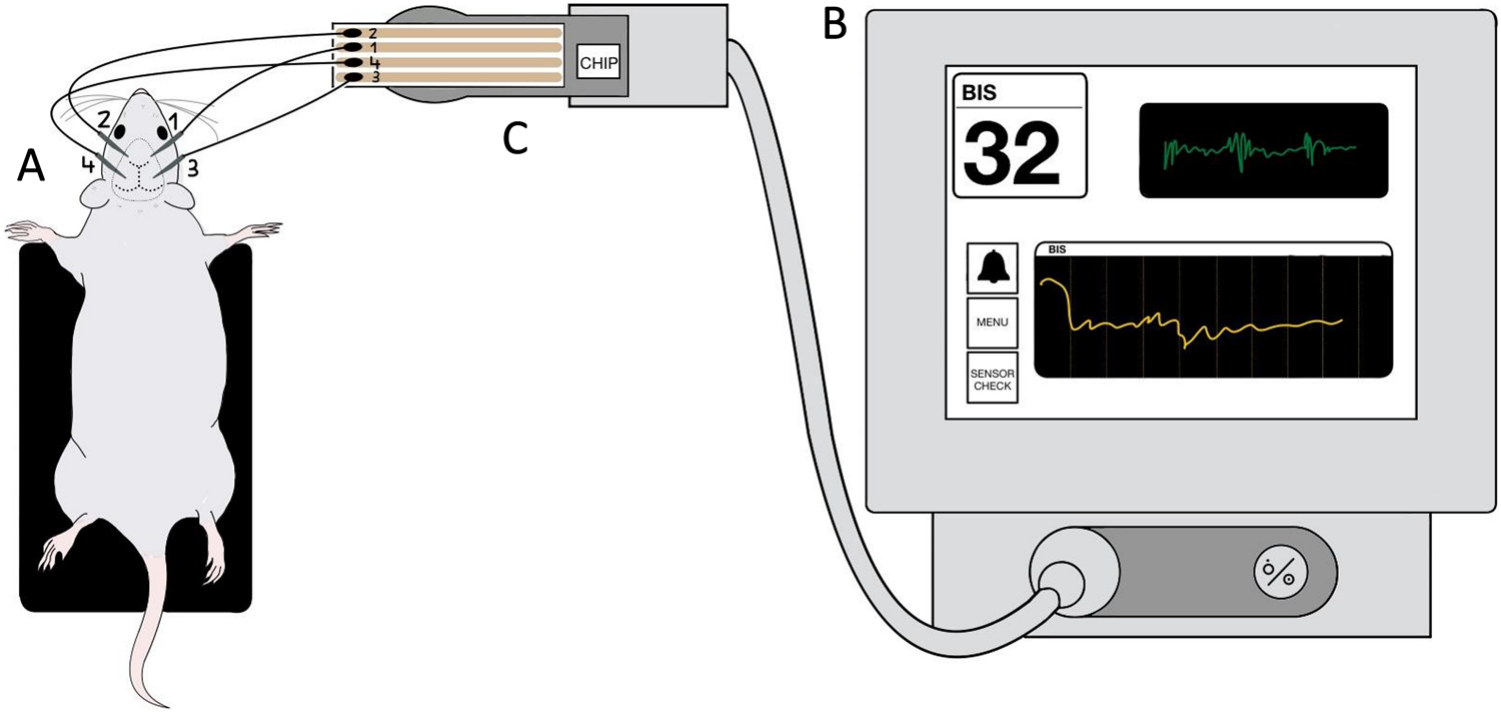
Experimental setup showing the subdermal needle electrodes on the mouse scalp, the BIS™ monitor and the sensor-interface. **A** The anesthetized mouse is placed on the heating pad with the subdermal needles inserted on the scalp. **B** The monitor shows the raw EEG signals (upper right window), the BIS™ index with a range between 0 and 100 (upper left window) and a trend development of the BIS™ index along with a selectable secondary variable (lower window). **C** The EEG signals from the electrodes reach the BIS™ monitor through the custom-made sensor interface

Four subdermal needle electrodes were inserted into the scalp of the mouse (Fig. 1). Two electrodes (BIS™ sensor numbers 1 and 2) were placed above the left and right frontal lobes and the reference and ground electrodes (BIS™ sensor numbers 3 and 4) were placed above the left and right occipital lobes. The mean duration of anesthesia for the complete preparation before starting the recordings was 10 min. To observe concentration-dependent changes in the EEG and the indices provided by the monitor, 3 different concentrations of isoflurane were applied cyclically throughout the process with a flow rate of 192 ml/min. The experiment was started with 1.2% isoflurane. After 7 min, the isoflurane concentration was increased to 2.2% for 5 min (longer periods lead to very low breathing rates of 20–30 breaths/min at 2.2%). This anesthesia cycle was repeated one time and then terminated with 1.6% isoflurane for 5 min. During the whole procedure, surgery level of the anesthesia was tested with the standard paw pinch test every 30 s.

### Data analysis

Data analysis was performed using native toolboxes and custom scripts in MATLAB-R2019a. The raw EEG signals were converted to vectors stored in the.mat format from r2a file format and were divided into different segments relating to different concentrations. The time series for BIS™ index and suppression ratio were extracted for every concentration with a temporal resolution of 1 s.

All EEG analyses were focused on suppression ratios and suppression durations with respect to the changing isoflurane concentrations. A burst-suppression detection algorithm (BSA) was developed to classify bursts and suppressions in the raw EEG signals. The BSA was designed to detect bursts and suppressions based on visual and subjective amplitude thresholds and delivered binary series in the form of ONEs (bursts) and ZEROs (suppression). Any signal above the amplitude threshold was classified as a burst and any signal below the amplitude threshold was classified as a suppression episode. Along with the amplitude threshold an additional temporal threshold was included in the BSA to classify any isoelectric signal which lasted for more than 0.5 s as one bout of suppression [34]. Though BSA was used to show the ease of burst suppression detection at these anesthetic dosages, the algorithm was verified by comparing the resultant binary series with that of a traditional nonlinear energy operator (NLEO) [35] which is widely used to detect bursts and suppressions. Instantaneous energy in the raw EEG signal was assessed to detect spikes [36] and bursts were identified at or above one standard deviation. The resultant binary series of BSA and NLEO across all concentration cycles across all mice were compared using Pearson correlation coefficient.

For the following analyses, only the EEG signals during the last 3 min of every concentration were selected to avoid metabolic adaptation artifacts after changing anesthetic concentrations. Using the resultant binary series, suppression durations were calculated for individual concentrations by counting the number of zeros in every suppression segment. To compare suppression durations at different isoflurane concentrations, the time series for suppression durations were calculated with a moving mean (window length of 10 consecutive suppressions) across all the concentrations for all the mice.

Suppression ratios were calculated according to the standard method of the BIS™ monitor, i.e., the percentage of suppressed EEG signals in the previous 63 s [34]. Burst ratios were calculated as the percentage of bursts in the previous 63 s of the EEG signals. Additionally, across all the concentrations, means of the calculated suppression ratios based on the BSA were compared with the means of the real time suppression ratios provided by the monitor to estimate the difference in burst-suppression detection between the monitor and the BSA.

To estimate the concentration dependent trend of the BIS™ index, the mean BIS™ index for every isoflurane concentration was calculated for each mouse from the real time BIS™ indices provided by the monitor.

To estimate state entropy (referred to as spectral entropy in this article) in anesthetized mice, 7 short chunks of data without burst suppression patterns were found with visual inspection in 3 out of 9 mice pooled together. These chunks of EEG signals were looped on the computer to generate 7 sets of EEG signals each of which were 1 min long. These EEG signals were replayed to the GE ENTROPY™ module (GE Healthcare, Chicago, Illinois, USA) through the computer [37]. The spectral entropy indices for these sets of data were extracted to identify the range of entropy values for anesthetized mice.

### Statistical analysis

All statistical tests and data plots were performed using GraphPad.Prism.9.3.1.471 (GraphPad Software, San Diego, California USA) except for the Durbin & Skillings–Mack test which was performed on XLSTAT v24.3.1342 (Addinsoft, Paris, France). Hence, for the comparison of suppression ratios and burst ratios across all the 3 concentrations on 9 mice, the Durbin & Skillings-Mack test was performed, which is a paired nonparametric test for repeated measures with missing values. The same comparison was tested with 7 mice (after removing the 2 mice with missing values) using the Friedman test and a post hoc Dunn’s multiple comparison test. The comparison of suppression ratios in 9 mice calculated by the monitor and the BSA was statistically tested with the Wilcoxon signed-rank test. The comparison of concentration dependent BIS™ index delivered by the monitor was statistically tested with Friedman test and a post hoc Dunn’s multiple comparison test on 8 mice (after removing the mouse with the missing data at 1.6% isoflurane). The summary data across mice for each variable are presented as medians with interquartile range. The statistics are reported at 95% confidence interval (*P*-value < 0.05). The absolute *P*-values digits are reported for every statistical test.

## Results

For two animals, the 1.2% and the 1.6% isoflurane concentration steps were excluded respectively, due to signs of movement (subtle toe movements) and prolonged respiratory depression (absence of breathing above 10 s).

**Fig. 2:**
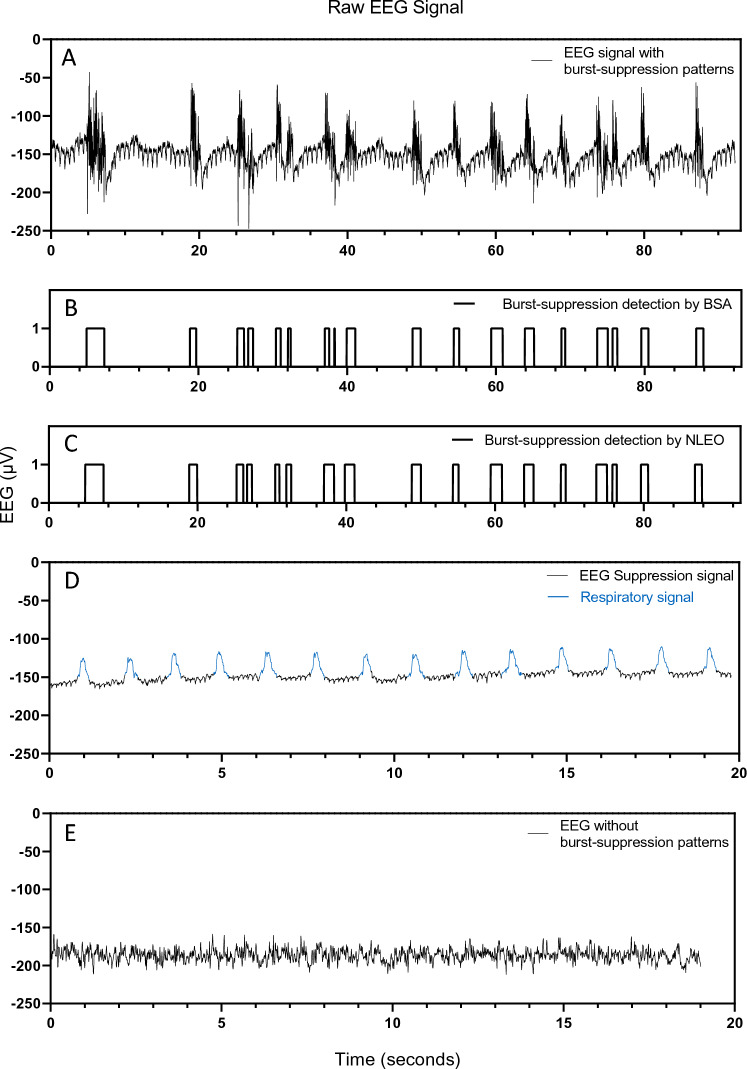
Raw EEG signals recorded with subdermal needle electrodes during surgical anesthesia in mice. **A** Burst suppression patterns in the EEG signals. **B** Burst suppression patterns in **A** detected with the BSA and transformed into binary series with ZEROs (suppression) and ONEs (burst). **C** Burst Suppression patterns in **A** detected with NLEO and transformed into binary series with ZEROs (suppression) and ONEs (burst). **D** Suppression EEG signal along with the embedded respiratory signals (in blue). **E** EEG signal without burst suppression patterns

The signals collected through subdermal needle electrodes were observed on the BIS™ monitor as raw EEG signals (Fig. 2A). All the tested isoflurane concentrations predominantly show burst suppression patterns. Moreover, the BSA could efficiently transform the raw EEG signals into binary series of bursts and suppressions (Fig. 2B). The burst suppression detection of the NLEO matched the results of BSA (Fig. 2C) and the average Pearson correlation coefficient between the two resultant binary series across all cycles across all mice was 0.75. The raw EEG signals also contain respiratory signals which are evident during suppression periods (Fig. 2D). 3 out of 9 mice had small sections of EEG signals without burst suppressions (Fig. 2E). Concentrations lower than 1.2% isoflurane were not sufficient to keep the mice unresponsive to paw pinch.

### Suppression durations

**Fig. 3:**
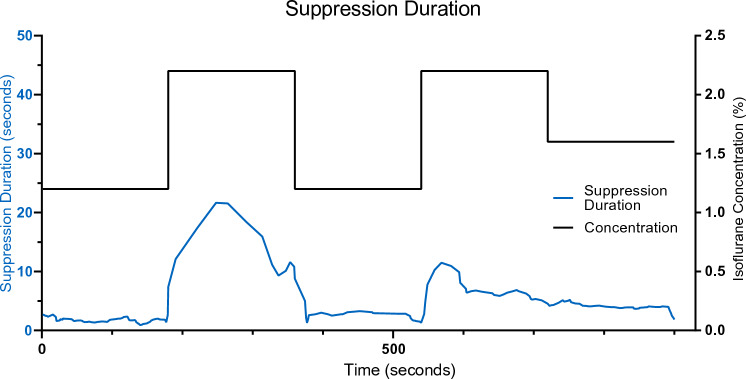
Suppression durations from an individual recording at different isoflurane concentrations (blues line). The EEG recordings were acquired at isoflurane concentrations of 1.2%, followed by 2.2% (black line). The cycle was repeated and the experiment was concluded by the application of 1.6% isoflurane. Corresponding suppression durations represented as the moving mean of 10 consecutive values are positively correlated with the concentration curve

A concentration-dependent change in suppression duration was observed in the raw EEG signals on the BIS™ monitor (Fig. 3). In all the 9 mice, suppression durations increased with the increase in isoflurane concentration.

### Burst/suppression ratios

**Fig. 4:**
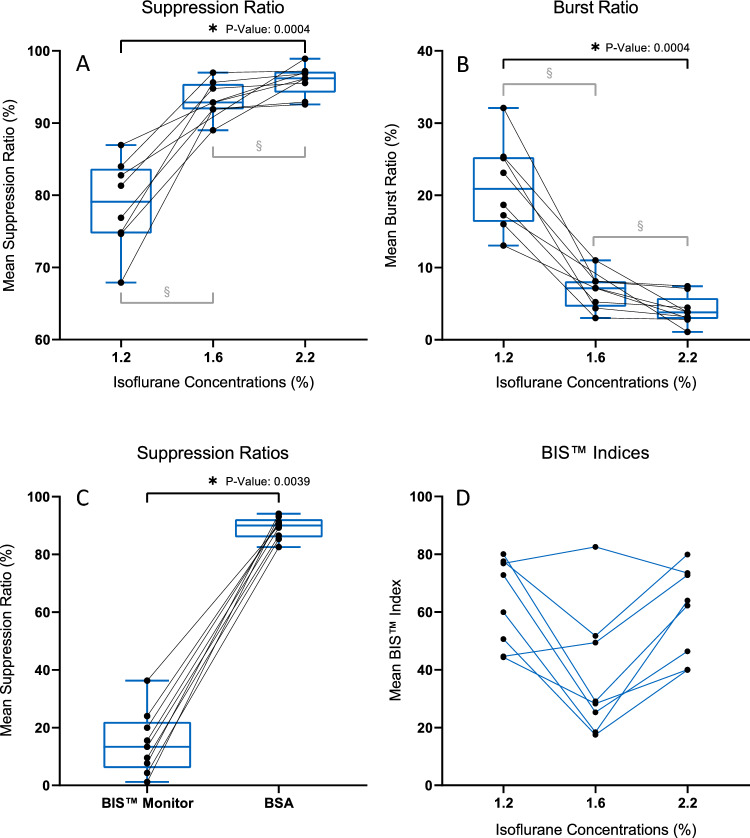
Suppression ratios and Burst ratios during GA in mice. **A** Mean suppression ratios increased from isoflurane concentrations of 1.2% to 1.6% to 2.2% for all mice. **B** Mean burst ratios decreased from isoflurane concentrations of 1.2% to 1.6% to 2.2% for all mice. **C** The mean suppression ratio provided by the BIS™ monitor was significantly lower than the mean suppression ratio calculated by the BSA. **D** Mean BIS™ indices during GA at different isoflurane concentrations. BIS™ indices were not correlated with the concentration of isoflurane. 6 out of 8 mice showed decreasing BIS™ indices when isoflurane concentration was increased from 1.2 to 1.6%. However, the BIS indices increased for all the mice when the concentration was increased from 1.6 to 2.2%

The suppression ratios increased and the burst ratios decreased with the increase in isoflurane concentrations in all 9 mice. The Skillings-Mack test showed that the paired groups for different isoflurane concentrations have significantly different (α = 0.05, P-value ≤ 0.001) suppression ratios (Fig. 4A) as well as burst ratios (Fig. 4B). The post hoc multiple comparison correction (Friedman test with Dunn’s multiple comparison, α = 0.05) showed significant increase (corrected *P*-value < 0.001) in suppression ratios and significant decrease (corrected *P*-value < 0.001) in burst ratios from 1.2 to 2.2% isoflurane. However, the group comparisons of 1.2% v/s 1.6% and 1.6% v/s 2.2% isoflurane concentrations did not show significant differences (corrected *P*-value = 0.184) in both suppression ratios and burst ratios after post hoc multiple comparisons.

The mean suppression ratio per mouse throughout the experiment calculated using the BSA was not representative of the mean of the real time suppression ratios provided by the BIS™ monitor. The BIS™ suppression ratios for all the mice were significantly lower (Wilcoxon signed-rank test, *P* value = 0.004) than the BSA suppression ratios (Fig. 4C). The BIS™ monitor underestimated the absolute ratio of suppressed EEG by 83.6%.

### BIS™ index

The BIS™ indices from the monitor were irregular and did not show consistent negative correlation with the increasing isoflurane concentrations in mice (Fig. 4D). The group means of BIS™ indices for different isoflurane concentrations were not significantly different (Friedman test with Dunn’s multiple comparison, α = 0.05, *P*-value = 0.120).

### Spectral entropy index

**Fig. 5:**
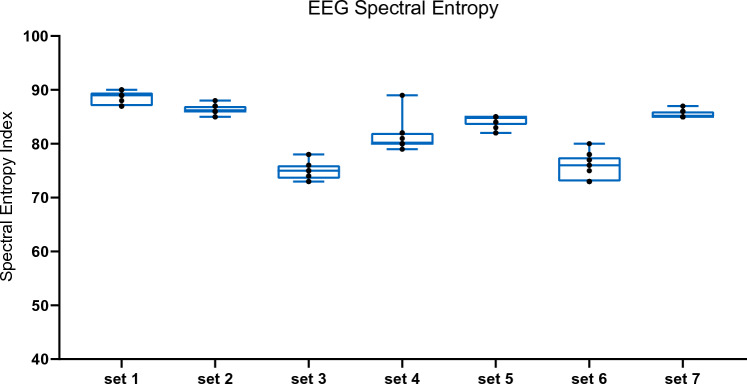
Spectral entropy index of replayed non-burst-suppression EEG signals. From the raw EEG signals pooled from all the mice, 7 sets of EEG signals without burst suppression patterns were selected, looped for 1 min each and were replayed to the GE Entropy™ module through a computer. The monitor generated spectral entropy indices which ranged from 70 to 90 across all the 7 sets

The spectral entropy indices across the 7 sets of EEG signals without burst suppression from the 3 sampled anesthetized mice ranged from 70 to 90 (Fig. 5).

## Discussion

In the present study, we introduce a simple and novel way to monitor brain function during GA in mice using subdermal needle electrodes and a modified clinical brain function monitor. Although other groups have used subdermal needle electrodes and conductive, adhesive gel patches on the scalp to record EEGs in freely moving rats [38, 39] and mice [40], there are hardly any applications so far to monitor intra-operative anesthesia administration in mice using EEG acquisition systems.

The concept of MAC to navigate GA is well known. MAC was initially formulated as an index for comparing anesthetic potency based on pharmacokinetics of inhaled anesthetics and the motor response of subjects under GA [41]. In other words, MAC is defined as the minimum alveolar concentration of an inhaled anesthesia, required to prevent movement in 50% of subjects to a surgical stimulus [42]. It is often underestimated that MAC more closely resembles anesthesia of the spinal cord rather than the brain [43] as minimum concentrations to suppress movement increase nearly threefold when anesthesia is specifically delivered to only the brain and not the spinal cord [44].

These circumstances may lead to inconsistent and inappropriate levels of anesthesia for surgeries, especially when different MAC values are consulted from various rodent models [45–47]. The anesthetic potency varies also among inbred, outbred, wild, and laboratory mouse strains [46, 48]. The potency of volatile anesthetics is also known to change with age in humans and animals [49–51]. Together, this can result in a wide range of anesthesia concentrations used for rodent experiments [46].

All anesthetics influence the brain, either at subcortical or at cortical target areas [52, 53]. While other methods of anesthetic monitoring may indirectly provide information on the brain under anesthesia, electroencephalographic (EEG) monitoring directly monitors the activity of the brain, the target organ of GA. It is becoming increasingly recognized that EEG-based monitoring of the anesthetized brain has the potential to transform monitoring “depth of anesthesia” into providing “quality anesthesia” [54]. The present study showed that homogeneous and directly controlled administration and application of GA via brain function monitors provide such a quality of anesthesia.

Human EEG monitoring devices have been used to monitor the level of anesthesia in several species such as in pigs [55], goats [56], dogs [57, 58], rabbits [59, 60], birds [61], horses [62] and dolphins [63]. Despite adequate representation of raw EEG signals, the output parameters and indices of the BIS™ monitor did not directly translate to meaningful information while monitoring the mouse EEG under isoflurane anesthesia. The BIS™ algorithm is designed for humans and the microanatomy of frontal cortex and subcortical structures differs partly between primates and rodents [64–66]. In humans, inhalational anesthetics are known to have an inverse relationship with BIS index [67–70] but with increasing anesthetic concentrations, the reductions in BIS index reaches a plateau at around 40 [70, 71]. Human studies also reported a paradoxical increase in BIS index during increasing concentrations of isoflurane, which is thought to be related to high-frequency pre-burst EEG activity [72, 73]. A paradoxical increase of the BIS index (> 60) was also reported from dogs [57, 74] and cats [75] following increased alveolar concentrations of isoflurane. Our results are in line with these results showing the paradoxical increase in BIS index at higher isoflurane concentrations (2.2%). The dominance of respiratory signals during long suppression periods at higher isoflurane concentrations (2.2%) may also have contributed to misleading BIS™ indices.

Spectral entropy of an EEG can be used as a measure of hypnosis during anesthesia [76]. State entropy is a spectral entropy parameter computed from 0.8 to 32 Hz. These entropy indices range from 0 to 91. An index that is equal to 91 means that the subject is fully awake and a state entropy of 0 means the EEG signal is isoelectric with no brain activity [77]. For humans, the recommended range of spectral state entropy index during GA is between 40 and 60 [33], providing quantitative and qualitative measurement of the level of anesthesia [78]. It was also shown that entropy monitoring is as reliable as BIS monitoring [79] for anesthesia titration. The clinically relevant target range for BIS and state entropy values during GA in humans is 40–60 [33]. On the contrary, with a small set of samples of non-burst suppression EEG signals deriving from our recordings, we found that the state entropy in mice ranges from 70 to 90. The present study was not designed to evaluate appropriate spectral entropy indices for an adequate mouse anesthesia, although consistent results from our small sample size point towards a potential establishment of meaningful indices for mice and other rodents.

Similarly, even clinical/human studies have shown inaccuracies in automated detection of neurophysiology for these devices. It is known that the device underestimates EEG suppression ratios as compared to suppression ratios derived from visual analysis of intraoperative EEG [80, 81]. We could show that the suppression ratios in mice were also underestimated by the BIS™ monitor as compared to the suppression ratio calculated by our BSA method, which is based on visually estimated thresholds. Clearly, the “contamination” of the suppression EEG signals by respiratory artifacts in our data led to sub-optimal detection strategies because of artificial contribution of these super-threshold amplitudes for the suppression detection algorithm of the BIS™ monitor [82].

Isoflurane is the most frequently administered anesthetic for surgical interventions in mice [83, 84] and EEG signals at surgical isoflurane concentrations predominantly show isoelectric and discontinuous burst suppression patterns [85]. With the support of a simple and cost-effective setup established in the present study, researchers can visually monitor the anesthetic level which is crucial to minimize the suppression duration while maintaining a stable anesthetic state. Using the presented method, the signals can also be recorded and stored for further analysis. It is important to note that the respiratory rate is also embedded in the signals which is an informative parameter that can be used later to compare the intraoperative physiologic status across mice. Although the present study was focusing on isoflurane anesthesia in mice, the present approach can be easily adapted to other rodents and other volatile anesthetics. Moreover, the technique can be used to minimal-invasively record simple EEG signals during the administration of intravenous anesthetics as well.

The 3R (Replacement, Reduction, Refinement) principle was formulated to provide a framework for a humane treatment of animals in research [86, 87]. The present study strongly supports reduction and refinement. By an appropriate anesthetic administration, lethality due to an anesthetic overdose can be minimized.

The optimized and well-controlled level of anesthesia also results in a robust cohort of experimental animals with a reproducible impact on cognition and behavior. Such refinements in animal experimentation and animal welfare strongly supports using these in vivo technologies as a standard. The techniques presented here are minimally-invasive as well as easily adaptable in a variety of experimental approaches and surgeries. The procedure can be performed both in prone and supine positions without a stereotactic frame. One might have to secure the subdermal needles with temporary adhesive tapes while the mouse is supine. Either the interface between the subdermal needles and the BIS™ monitor can be custom made or interfaces that are previously established for clinical purposes could be applied [88].

## Limitations of the method

Surgeries in supine positions and especially repositioning of the animal from supine to prone positions during surgery may cause a loss of the EEG signal through displacement of the needle electrodes. In several trials we could not avoid this, even though we secured the electrodes with medical tape to the head. Brief readjustment of the electrodes after moving the animal is recommended. Implantations of complex neuronal electrode systems may demand free access to the skull so space for additional needle electrodes could either be very limited or the needle electrodes may not be placed on the skull of the animal at all. For these situations our method might not be practical. First-time users may lack the experience to read and analyze a raw EEG-signal online during surgeries. Inadequate general anesthesia at sub-MAC levels or only partly introduced LOR may lead to spontaneous activities of skeletal muscles such as masticatory muscles, contaminating the EEG signal.

We suggest investing some time in learning how a typical EEG signal looks during several vigilance states such as wakefulness, GA and burst suppression.

## Conclusion

A patient monitoring system designed for human EEG monitoring can be applied to monitor mouse EEG during anesthesia by replacing clinical EEG sensors with subdermal needle electrodes. The suppression duration in the raw EEG signals on the monitor can be used as a visual cue for avoiding excessive anesthesia administration during surgery. This method can be applied in a variety of surgical and manipulative situations in animal research, where a safe and well-monitored GA is desired. Moreover, the method could pave the way for generating reproducible studies in preclinical research by assuring stationary experimental conditions including the optimization of GA administration which in turn guarantees scientific quality of collected data in translational medicine.

## Declarations

### Authors statement

All authors agreed on the submitted manuscript and its latest form to be submitted.

### Patients statement

No patients or patient data were included in the present experiments.
